# Supplementary material for: The causal relationship between the human gut microbiota and pyogenic arthritis: a Mendelian randomization study
Source: Front Cell Infect Microbiol. 2024 Nov 26;14:1452480. doi: 10.3389/fcimb.2024.1452480 (PMC11629706; doi:10.3389/fcimb.2024.1452480)
Supplement: Supplementary file 1 [file DataSheet1.zip › mendelian test/ebi-a-GCST90027780.csv_funnel.pdf]

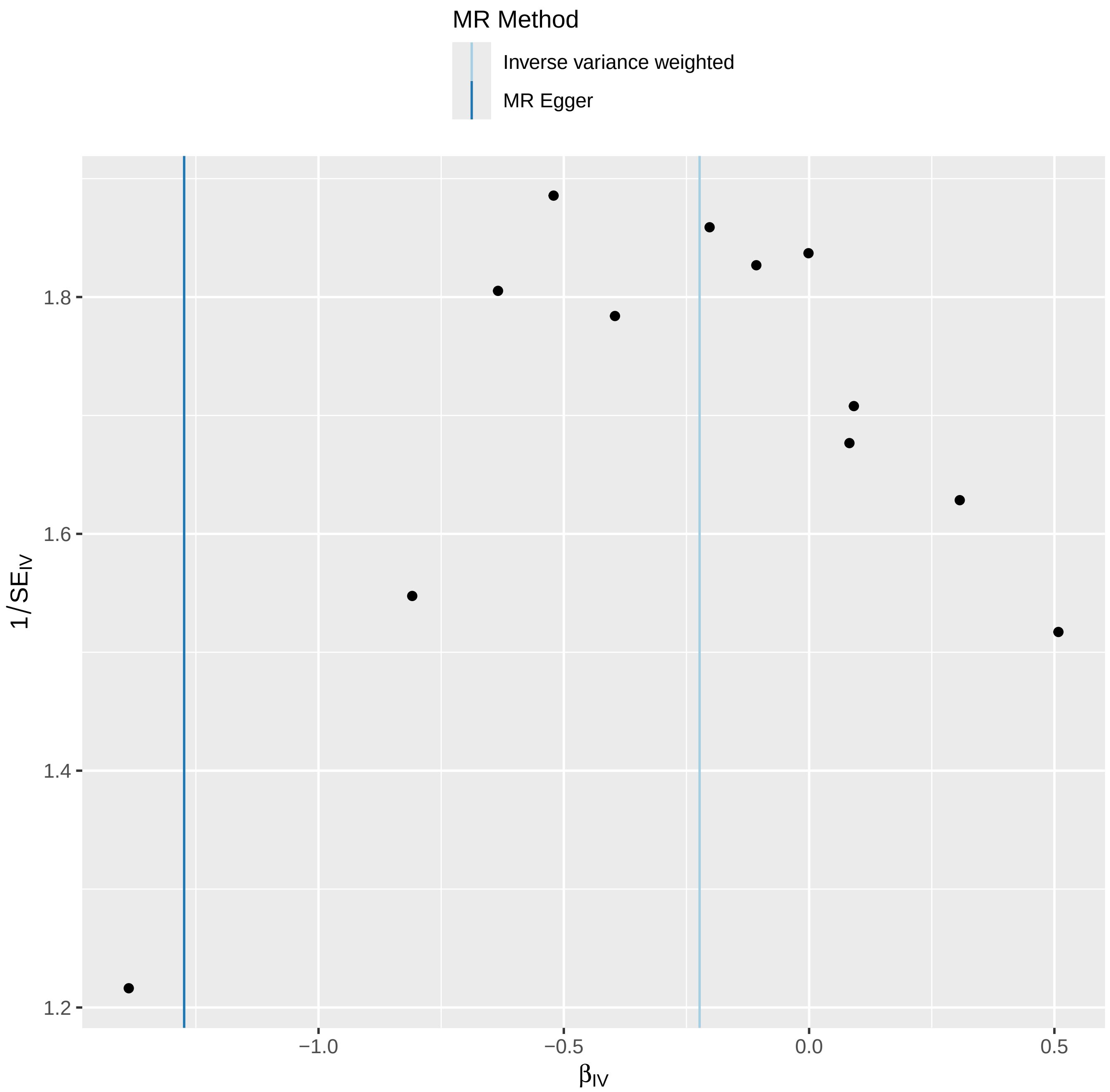
MR Method
Inverse variance weighted
MR Egger
1.8
1.6
1.4
1.2
1/SE$_{IV}$
−1.0
−0.5
0.0
0.5
β_{IV}
